# Supplementary material for: Anticipatory coadaptation of ankle stiffness and sensorimotor gain for standing balance
Source: PLoS Comput Biol. 2019 Nov 22;15(11):e1007463. doi: 10.1371/journal.pcbi.1007463 (PMC6897426; doi:10.1371/journal.pcbi.1007463)
Supplement: S2 Text — (DOCX) [file pcbi.1007463.s002.docx]

Anticipatory coadaptation of ankle stiffness and neural feedback for standing balance

Charlotte Le Mouel, Romain Brette

Supplementary Text 2: Pade approximation

The first order Pade approximation of the delay is given by:

$$e^{-X}=\frac{e^{-X/2}}{e^{X/2}}\approx\frac{1-X/2}{1+X/2}$$

The Pade approximation consists in approximating the function θ$\left( t-\tau_{delay} \right)$ by a function $\theta_{approx}\left( t \right)$ which follows:

$\frac{\tau_{delay}}{2}\dot{\theta}_{approx}\left( t \right)+\theta_{approx}\left( t \right)=\theta\left( t \right)- \frac{\tau_{delay}}{2}\dot{\theta}\left( t \right)$

Examples:

1. Suppose $\theta\left( t \right)$ is a step function defined by (Figure S2.1.A in black):

- $\theta\left( t \right)=0 for t<0$
- $\theta\left( t \right)=1 for t>0$

Then $\theta_{approx}\left( t \right)=1-exp(-\frac{2t}{\tau_{delay}})$ (Figure S2.1.A in red).

1. Suppose $\theta\left( t \right)$ is a sinusoidal function defined by $\theta\left( t \right)=\exp\left( i\omega t \right)$ (Figure S2.1.B, C, in black), then:

$$\theta_{approx}\left( t \right)= \frac{1-\frac{i\omega\tau_{delay}}{2}}{1+\frac{i\omega\tau_{delay}}{2}}\exp\left( i\omega t \right)$$

The amplitude is:

$$\sqrt{\frac{({1+({\frac{\omega\tau_{delay}}{2})}^{2})}^{2}}{({1+({\frac{\omega\tau_{delay}}{2})}^{2})}^{2}}}=1$$

The phase lag is:

$$\phi\left( \frac{1-\frac{i\omega\tau_{delay}}{2}}{1+\frac{i\omega\tau_{delay}}{2}} \right)= -2\arctan\left( \frac{\omega\tau_{delay}}{2} \right)$$

This corresponds to a time lag of $\tau_{delay}$ for small $\omega\tau_{delay}$ (Figure S2.1.C in red) and π for large $\omega\tau_{delay}$ (Figure S2.1.B in red).

Thus, the Pade approximation $\theta_{approx}\left( t \right)$corresponds to $\theta\left( t-\tau_{delay} \right)$ if $\theta$ changes slowly compared to $\tau_{delay}$ (Figure S2.1.C), whereas fast variations in $\theta$ are distorted (Figure S2.1.A, B).


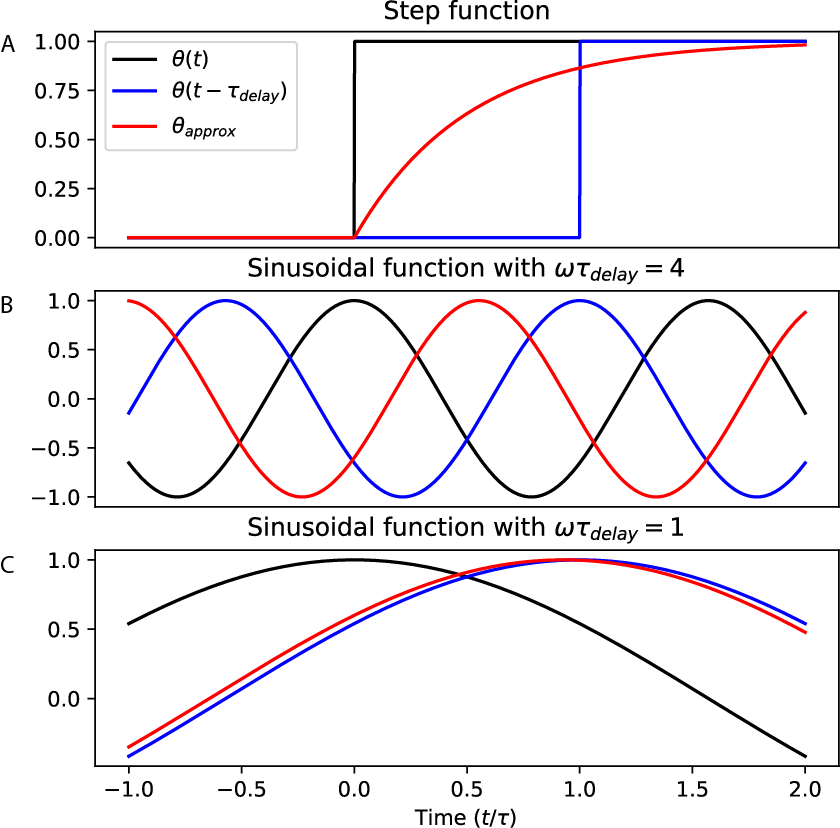


Figure S2.1 Pade approximation of the time delay.

A. Step function in black, delayed step function in blue and Pade approximation of the delayed step function in red. B-C Sinusoidal function with $\omega\tau_{delay}=4$ and 1 (respectively for panels B and C) in black, delayed sinusoidal function in blue and Pade approximation of the delayed sinusoidal function in red.
